# Socioeconomic gradients in general and oral health of primary school children in Shiraz, Iran

**DOI:** 10.12688/f1000research.8641.1

**Published:** 2016-04-27

**Authors:** Ali Golkari, Aira Sabokseir, Aubrey Sheiham, Richard G. Watt

**Affiliations:** 1 Department of Dental Public Health, School of Dentistry, Shiraz University of Medical Sciences, Shiraz, 71345-1836, Iran; 2 Research Department of Epidemiology and Public Health, University College London, London, WC1E 7HB, UK

**Keywords:** Health status disparities, Inequalities, Oral health, Socio-economic factors

## Abstract

**Background: **Health status is largely determined by socio-economic status. The general health of individuals at higher social hierarchy is better than people in lower levels. Likewise, people with higher socio-economic status have better oral health than lower socio-economic groups. There has not been much work regarding the influence of socio-economic status on the health conditions of children in developing countries, particularly in Iran. The aim of this study was to compare the oral and general health conditions of primary school children of three different socio-economic areas in the city of Shiraz, Iran.

**Methods: **This cross-sectional study was conducted on 335, 8- to 11-year-old primary schoolchildren in Shiraz. The children were selected by a three-stage cluster sampling method from three socio-economically different areas. Tools and methods used by the United Kingdom’s Medical Research Council were used to obtain anthropometric variables as indicators of general health. The Decay, Missing, Filled Teeth (DMFT) Index for permanent teeth, dmft Index for primary teeth, the Modified Developmental Defects of Enamel (DDE) Index, the Gingival Index (GI) and the Debris Index-Simplified (DI-S) were used for oral health assessment.

**Results: **Height (P<0.001), weight (P<0.001), and BMI (P=0.001) significantly increased as the socio-economic status of area increased. GI score (P<0.001), DI-S score (P<0.001), number of permanent teeth with DDE (P=0.008), and number of DDE lesions in permanent teeth (P=0.008) significantly decreased as the socio-economic status of area increased.

**Discussion: **Findings of this study generally confirmed that social gradients exist in both general and oral health status of the primary schoolchildren of Shiraz. The influence of socio-economic status on health condition means children have different life chances based on their socio-economic conditions. These findings emphasize the significance of interventions for tackling socio-economic inequalities in order to improve the health status of children in lower socio-economic areas.

## Introduction

Health status is largely determined by social class and socio-economic status. Understanding the association between socio-economic status and health outcomes is of great importance for planning health promotion strategies. It is generally accepted that “there is a social gradient in health” ^1– 4^. In the national trend, the gradient is the status of an individual in socio-economic hierarchy and shows that people at the top of social hierarchy have better health than those in lower levels ^5^. In the international trend, the gradient indicates more affluent countries have better health outcomes compared to poorer countries ^6^.

The effect of socio-economic status is shown on a wide range of health outcomes from drug misuse related diseases ^7^ to age-specific mortality ^8^. A study on 6 to 11 year-old children of the United States found that social class had positive association with minor and major physical disorders such as colds, infections, allergies, diabetes, and even epilepsy ^9^. The relationship between household income and a range of health outcomes in children and adolescents has been shown in Britain ^10^. A study in France has found a significant positive correlation between family income and some of the most important child health indicators such as anthropometric measurements ^11^.

In consistence with general health, social gradients are shown in oral health indicators. It seems that despite the remarkable improvements in the averages of oral health indices among communities in the last decades, inequities in oral health - mostly related to social inequalities - continue to exist ^12, 13^.

A study on the relationship between work conditions and health inequalities in Switzerland has found clear social gradients for almost all adverse working status and the outcomes of oral health ^14^. In a case-control study in Mexico, lower socioeconomic status was identified as a risk factor for non-syndromic orofacial clefts ^15^. Another study on Chinese 5-year-old children found that there were notable gradients in carious primary teeth related to their household income ^16^. A study on young children of Salem, Tamil Nadu, India, showed that those from lower socio-economic groups, especially whose parents' had lower education were more likely to suffer from Early Childhood Caries ^17^. It was also shown that poor socio-economic background can be a strong predictor for poor oral hygiene ^18^.

There has been limited evidence in Iran regarding the association between socio-economic status and general and oral health gradients, particularly among children. Therefore, this study was designed to assess the existence of social gradients in oral and general health of 8- to 11-year-old primary school children in three different socio-economic areas of Shiraz, a city in Southern Iran.

## Material and methods

This cross-sectional study was conducted on 8- to 11-year-old children (third to fifth grade primary school children) of the city of Shiraz, Iran, in 2009. Approval and ethical permission were obtained from the National Ethical Committee in Medical Research of Iran (# 85/p/3/1095). Permission to enter the selected schools was then obtained from the Educational Head Office of the Province of Fars (# 17568/55). Based on the prevalence of Developmental Defects of Enamel (DDE) and a confidence level of 95%, a sample size of 335 was estimated appropriate for this study.

A three-stage cluster sampling approach was used to select the children. The main Educational Affairs Office of Shiraz had a category which divided the city into three areas based on socio-economic status: upper, middle, and lower social class areas. This kind of division seemed more appropriate for this study than any other division, such as geographically separated zones. One boys' and one girls' school (primary schools are segregated in Iran) were selected from each of the three socio-economically different areas. This was done by simple randomization using the list of primary schools in each area. Then, two classes were randomly selected in each school from the third, forth, and fifth grades. All children present in each selected class were included in the study, only if their parents provided written consent.

Although socio-economic differences between the three areas were well recognized by officials (Educational Head Office 2006), the number of parents having an occupation (any job), and the number of parents having a permanent occupation (as a job security indicator) were used as socio-economic related variables to compare the three areas and confirm the socio-economic difference between children chosen from these areas ^19, 20^. Related information was obtained from schools' administration offices.

Height and weight are valuable indicators of present and past health status. Dividing the body height into trunk and leg length provides more precise information particularly on nutrition status of the early years of life ^21, 22^. Standing height, leg length, weight, and BMI (Body Mass Index) were used in this study as indicators of general health.

To obtain children's height and weight, the tools and methods used by United Kingdom’s Medical Research Council were adopted ^22^. For weight, a digital scale (Beurer Electronic Weight Scale PS 07, Germany) with accuracy of 100 grams was used. It was calibrated in each school. A wall mounted height meter with accuracy of millimetres was used for measuring children's standing height. A similar metre adjusted to the seat of a chair was used to measure the length of the upper trunk, from head to the chair’s seat (sitting height). The leg length was calculated by deduction of the sitting height from the standing height. BMI was calculated by dividing the weight by the square of the standing height. Identically calibrated tools were used for all cases in all schools.

Assessment of dental caries, DDE and gingival health determined the oral health status of children. Clinical intra-oral examinations were carried out using WHO screening criteria to record data on Decay, Missing, and Filled Teeth (dmft) for the primary dentition and DMFT for the permanent dentition ^23^. Permanent teeth were assessed from first molar to first molar in each arch. Therefore, second permanent molars, if present, were not assessed. DDEs of permanent teeth were recorded based on the Modified DDE Index ^24^. Gingival health and oral hygiene were assessed using the Gingival Index (GI) ^25, 26^ and the Debris Index – Simplified (DI-S) ^27^. All examinations were carried out in classrooms using natural light, disposable mirrors and tongue blades.

Normality of distribution of each outcome variable was assessed by a histogram. All continuous variables had a distribution close to normal. The difference between sexes was analysed by a t-test. Two socio-economic variables of number of parents with any occupation and with a permanent occupation were compared among the three areas using one-way ANOVA. Anthropometric measurements, gingival health, and oral hygiene status were also assessed among the three areas by one-way ANOVA. Anthropometric measurements (linear regression), gingival health status, and oral hygiene status (quantile regression) were also compared among the three areas after adjusting for sex and age. DMFT, dmft, number of teeth needed treatment, number of permanent teeth with DDE and total number of DDEs in children’s permanent dentition were compared between areas using Poisson regression model after adjusting for sex and age, and number of permanent teeth present in the mouth. SPSS statistical package (version 14) and STATA statistical package (version 10 Intercooled) were used. The significance level was set at α=0.05.

## Results

Consent forms were sent for 376 parents, out of which 335 accepted to participate (response rate = 89.1%). Table 1 shows the number of cases examined in each area by sex.

**Table 1.** Sample size and parents' occupation status of children by area and sex (N=335).

Area (socio- economic level)	Sex	Number of examined children	Mean number of parents with any occupation	Odds ratio of having at least one jobless parent	Mean number of parents with a permanent job	Odds ratio of having no parent with a permanent job
**Low**	**Girls**	69	1.76	0.08	1.55	< 0.0001
	**Boys**	43				
**Middle**	**Girls**	47	1.37	0.45	1.01	0.12
	**Boys**	54				
**High**	**Girls**	61	1.17	1	0.70	1
	**Boys**	61				
**Significance level of difference** **among areas**			P < 0.001		P < 0.001	

The number of parents with an occupation (P < 0.001) and with a permanent occupation (P < 0.001) significantly increased from the lower socioeconomic to the higher socioeconomic area ( Table 1). These results confirm that the three areas were socio-economically different.

Girls were slightly heavier and taller, but had shorter leg length than boys. All anthropometric variables had distributions close to normal. Children in lower socio-economic neighbourhoods were shorter and lighter, and had shorter legs. Even after adjustments were made for sex and age, still all anthropometric variables: height (P < 0.001), weight (P < 0.001), BMI (P = 0.001) and leg length (P < 0.001) of children significantly increased as the socio-economic status of area increased. These findings demonstrated clear social gradient in the general health status among Shiraz primary school children.

The average number (± standard deviation) of present deciduous teeth was 6.2 (± 4.3). Number of permanent teeth present in the mouth ranged from four to 24 with an average of 16 fully erupted permanent teeth per child ( Table 2). Number of present permanent teeth was significantly higher in girls (mean = 18.1) than in boys (mean = 13.6). The difference remained statistically significant after adjustment for age (P < 0.001).

**Table 2.** Permanent teeth, permanent teeth with DDE, and DDEs per child, by socio-economic areas.

Area	Average number of fully erupted permanent teeth per child	Average number of permanent teeth with DDE per child	Percent of permanent teeth with DDE (%)	Average number of DDEs per child
**Low-socioeconomic**	15.98	1.72	10.8	1.76
**Middle-socioeconomic**	15.35	1.19	7.8	1.26
**High-socioeconomic**	16.78	1.02	6.1	1.07
**Average/total**	16.01	1.30	8.1	1.36
**Significance level (1)**	--	P = 0.018		P = 0.025
**Significance level (2)**	--	P = 0.008		P = 0.008

(1) Adjustments made only for number of permanent teeth. (2) Adjustments made for number of permanent teeth, sex, and age.

Forty nine percent (164) of children had a DDE. There was no difference between the percentages of boys and girls having a DDE in permanent teeth. Table 2 shows the number of permanent teeth with DDE and the total number of DDEs in permanent teeth. Both variables increased as socio-economic status of area decreased. The trends were statistically significant for number of permanent teeth with DDE (P = 0.018) and the total number of DDEs in permanent teeth (P = 0.025) after adjusting for number of present permanent teeth in the mouth. The significance levels increased (P = 0.008 for both variables) after adjusting for age and sex in the regression model.

The average dmft (of the primary dentition) was 2.8 ± 2.5. Three quarters of the cases (75.2%) had a dmft of 1 or more. The average DMFT (of the permanent dentition) was 1.22 ± 1.5. Almost half of the sample (47.5%) had caries experience in their permanent teeth. DMFT increased from 0.7 in 8-year-olds to 1.4 in 11-year-olds. Although the mean DMFT was higher in girls (1.3) than in boys (1.0) (P = 0.02), the difference was not significant after adjustments for age and number of permanent teeth (P = 0.42).

There was no statistically significant trend in DMFT or dmft among areas. Children in the middle class area had the highest caries prevalence and highest need for treatment among all three areas. Although a clear path of social gradients was not observed, total number of teeth with treatment need was significantly lower in children in high socio-economic area compared with other children (P = 0.006). There was also a significant difference in mean number of deciduous teeth with treatment need between the high socio-economic area and the two other areas (P = 0.005) ( Table 3).

**Table 3.** DMFT, dmft, and number of teeth with treatment need, by socio-economic areas and sex.

Area	Sex	DMFT	Mean number of permanent teeth with treatment need	dmft	Mean number of deciduous teeth with treatment need	Total number of teeth with treatment need
**Low-** **socioeconomic**	Boys (N = 43)	0.56	0.33	3.0	2.9	3.2
	Girls (N = 69)	1.14	0.84	2.2	2.1	3.0
	Both (N = 112)	0.92	0.64	2.5	2.4	3.1
**Middle-** **socioeconomic**	Boys (N = 54)	1.54	1.13	4.7	4.2	5.3
	Girls (N = 47)	1.62	1.13	2.0	1.7	2.8
	Both (N = 101)	1.58	1.13	3.5	3.0	4.2
**High-** **socioeconomic**	Boys (N = 61)	0.97	0.54	3.4	2.5	3.0
	Girls (N = 61)	1.43	1.03	1.6	1.1	2.2
	Both (N = 122)	1.20	0.79	2.5	1.8	2.6
**Total (N = 335)**		1.22	0.84	2.8	2.4	3.2

A considerable number of children had gingival inflammation. Average GI score was 0.18 with only 82% of children being scored 0. The average DI-S was 0.52. The difference between the sexes was not statistically significant (P = 0.43 for GI and P = 0.44 for DI-S).

The gingival health significantly improved as the socio-economic status of area increased (P < 0.001). The significance did not change after adjustments for sex and age. There was also significant trend in oral hygiene status (P < 0.001). The number of children with dental plaque (P < 0.001), and also the average level of dental plaque in children (P < 0.001) decreased as the socio-economic status of area increased ( Table 4).

**Table 4.** Gingival health and oral hygiene status among the three socio-economic areas, by sex.

Area	Sex	Number of children with GI=0 (%)	Number of children with GI> 0 (%)	Average DI-S score
**Low-socioeconomic**	Boys (N = 43)	29 (67.4)	14 (32.6)	0.82
	Girls (N = 69)	53 (76.8)	16 (23.2)	0.70
	Both (N = 112)	82 (73.2)	30 (26.8)	0.75
**Middle-socioeconomic**	Boys (N = 54)	44 (81.5)	10 (18.5)	0.52
	Girls (N = 47)	36 (76.6)	11 (23.4)	0.50
	Both (N = 101)	80 (79.2)	21 (20.8)	0.51
**High-socioeconomic**	Boys (N = 61)	53 (86.9)	8 (13.1)	0.44
	Girls (N = 61)	60 (98.4)	1 (1.6)	0.19
	Both (N = 122)	113 (92.6)	9 (7.4)	0.32
**Total** **(N = 335)**		275 (82.1)	60 (17.9)	0.52
**Significance level of difference** **among areas**		P< 0.001		P< 0.001

Raw data Data on age, sex, socioeconomic, anthropometric and oral health variables are provided. Click here for additional data file. Copyright: © 2016 Golkari A et al. 2016 Data associated with the article are available under the terms of the Creative Commons Zero "No rights reserved" data waiver (CC0 1.0 Public domain dedication).

## Discussion

Findings of this study generally confirmed that social gradients exist in both general and oral health status of the primary school children of Shiraz. Children in lower socio-economic areas were shorter and lighter, and had shorter legs. In terms of oral health, socio-economic status had more effects on gingival inflammation, levels of plaque, and specially occurrence of DDEs, but was less related to dental caries experience and caries treatment need.

Most findings of this study were consistent with those of similar studies in other parts of the world. As an example, the social gradients seen in the anthropometric measurements were similar to those presented among French children ^11^. One of the strong relationships found in this study was between the area of living and the state of oral hygiene determined by DI-S. This finding was in accordance with the findings of a recent study by Mathur *et al.* in India ^18^. They had also divided a city, Delhi, into three socio-economically different areas and found that children in poorer areas were more susceptible for poorer oral hygiene.

In the current study, social gradient could not been shown in DMFT or dmft of studied children. Further research revealed that similar conditions had been reported by others. As an example, Sagheri *et al.* (2009) reported a social gradient in using preventive dental services among children of Ireland, but no such trend in DMFT of the same children ^28^. To justify such findings, one could blame the nature and shortcomings of the DMF Index or complexity of the risk factors for dental caries.

DDE is probably the most important oral health indicator assessed in this study. Presence of DDE on teeth can be an index of both general and oral health. Many early childhood adverse health conditions and diseases can increase the risk of developing a DDE on permanent dentition ^29^. Despite the importance of DDE as a health indicator, few studies have tried to assess the relationship of the prevalence of DDE, especially on permanent teeth, with socio-economic factors. One of such studies has been conducted by Basha *et al.* that reports a significant negative association between the presence of DDE and socio-economic status of children ^30^, a finding that is very similar to what is shown in the current study.

The influence of socio-economic status on health condition means children have different life chances based on their birthplace, or area of living, even inside one city. If they live in a higher socio-economic zone, they would have a higher chance for better oral and general health in comparison to their peers in neighboring lower socio-economic zones. These findings could help policymakers to make intervention decisions that might help to improve the health status of children in lower socio-economic areas.

Bearing in mind that no clear social class determinant or assessment tool exists in Iran, the authors decided to use the school area as the best available indicator of subjects' social class. Assessing the job status of parents acted as a proof that the selected groups of children were really from different backgrounds. Therefore, it seems fair to say that this study has been able to illustrate that oral health follows the other aspects of health in having gradients according to socio-economic status.

As oral health and general health follow the social gradients, tackling health inequalities would need identifying and understanding the underlying causes of problems ^31^. Upstream measures would be needed to build a society that could reinforce good oral and general health. Future assessment of the relationship between social gradients in oral health and general health is recommended.

## Conclusion

There are social gradients in general and oral health among primary school children of Shiraz, Iran. The gradient in oral health seems to follow the same pattern in general health.

## Consent

Written informed consent for participation in the study was obtained from the parents of the children.

## Data availability

The data referenced by this article are under copyright with the following copyright statement: Copyright: © 2016 Golkari A et al.

Data associated with the article are available under the terms of the Creative Commons Zero "No rights reserved" data waiver (CC0 1.0 Public domain dedication). 

F1000Research: Dataset 1. Raw data, 10.5256/f1000research.8641.d120612 ^32^.
