# Supplementary material for: Socioeconomic gradients in general and oral health of primary school children in Shiraz, Iran
Source: F1000Res. 2016 Apr 27;5:767. [Version 1] doi: 10.12688/f1000research.8641.1 (PMC4879935; doi:10.12688/f1000research.8641.1)
Supplement: Peer review history

# Peer review: Referee response for version 1

**DOI:** 10.5256/f1000research.9297.r13623

**Published:** 2016-05-25

**Authors:** Ali Kazemian

**Affiliations:** 1 Department of Community Oral Health, Mashhad School of Dentistry, Mahshad University of Medical Sciences, Mashhad, Iran

I found this article worth indexing. Well organized, well written.

I have read this submission. I believe that I have an appropriate level of expertise to confirm that it is of an acceptable scientific standard.

# Peer review: Referee response for version 1

**DOI:** 10.5256/f1000research.9297.r13930

**Published:** 2016-05-24

**Authors:** Myung Ki

**Affiliations:** 1 Department of Preventive Medicine, School of Medicine, Eulji University, Daejeon, Korea, South

The theme of this paper is, to my mind, very interesting (socioeconomic inequalities in general and dental health) and various general and dental measures with trustable definition are plus. The design and data analysis seem acceptable to support the conclusion. Thus, I believe that this paper merits indexation, but there is a few points to be addressed / discussed more.

  

1. For anthropometric variables, authors describe: “Even after adjustments were made for sex and age, still all anthropometric variables: height (P < 0.001), weight (P < 0.001), BMI (P = 0.001) and leg length (P < 0.001) of children significantly increased as the socioeconomic status of area increased.”
  However, no actual results on this appear in the table and please check in which table the result is shown. If this finding is less important and authors decided not to present the data, please indicate that “data is not presented”.
2. It is not clear why two p values are presented in the table 1 and I think it would be four p-values per each column. Similarly, only two p-values were presented in the table 2, instead of four as to four measures. I am sorry, if misunderstanding is mine.
3. For presentation of table 1, I wonder whether the use of Odds Ratio is proper approach. I think instead of OR, presenting different proportions for three area groups and statistical test with Chi-square would be relevant and complementary to other results in table 1, for example, Chi-square test for “having at least one jobless parent” for three area levels.
4. Use of area level SEP could be more justifiable, if the parents are actual residents of the areas to which their siblings attend, if additional information of this kind is available.

I have read this submission. I believe that I have an appropriate level of expertise to confirm that it is of an acceptable scientific standard.

# Peer review: Referee response for version 1

**DOI:** 10.5256/f1000research.9297.r13621

**Published:** 2016-05-16

**Authors:** Tayebeh Malek Mohammadi

**Affiliations:** 1 Oral and Dental Diseases Research Centre, Department of Dental Public Health, Kerman Dental School, Kerman University of Medical Sciences, Kerman, Iran

The manuscript reported very important subject in oral health' field, seems sound and very well written. It is acceptable for indexation. I suggest the authors should address some studies regarding oral health and general health and also oral health and socio-economic factors which have been reported in Iran.

I have read this submission. I believe that I have an appropriate level of expertise to confirm that it is of an acceptable scientific standard.
